# Effects of Compressed Air

**Published:** 1845-06

**Authors:** 


*Effects of Compressed Air.*—M. Triger, an engineer, having to work a mine in the vicinity of the Loire, in order to throw back the water, kept up a pressure of several atmospheres by means of a steam engine. The men working in air thus compressed to three atmospheres, experience at first pain, more or less intense, in the ears; this pain ceases as soon as the mercury in the manometre marks an altitude of three centimetres. Deglutition also at once dissipates it, owing, probably, to its carrying, through the Eustachian tube, to the central ear a certain proportion of air, which re-establishes the equilibrium of pressure on the tympanum from within and without. The intensity of the pain depends a great deal on the state of the health. Drunkenness always renders it intolerable.—*London Lancet.*

